# The influence of a single species on the space use of mixed-species flocks in Amazonian Peru

**DOI:** 10.1186/s40462-019-0181-5

**Published:** 2019-11-28

**Authors:** Sean M. Williams, Catherine A. Lindell

**Affiliations:** 1 Department of Integrative Biology, Michigan State University, East Lansing, MI USA; 2 Program for Ecology, Evolutionary Biology, and Behavior, Michigan State University, East Lansing, MI USA; 3 Department of Biology, College of the Holy Cross, Worcester, MA USA; 4 Center for Global Change and Earth Observations, Michigan State University, East Lansing, MI USA

**Keywords:** Anti-predation, Foraging behavior, Antbird, *Thamnomanes*, *Myrmotherula*, Rainforest, Habitat use, Multi-species group

## Abstract

**Background:**

The drivers of space use patterns of multi-species groups have been poorly studied, although mixed-species avian flocks are common throughout the world. In a mixed-species flock, multiple species move together and maintain proximity. The different species may or may not have conflicting preferences of space use. We hypothesized that the space use patterns of the flock are driven by a single species.

**Methods:**

We investigated the behavioral drivers of space use patterns of mixed-species flocks in Amazonian Peru by mapping 95% fixed-kernel home ranges of three flocks, which then we divided into high-use (inner 55% kernel utilization distribution) and low-use areas (lying outside the high-use area). We quantified the foraging and anti-predator behavior of individual birds in the flocks. We tested whether foraging and anti-predator behavior of different species were different in high use and low use areas of the flock.

**Results:**

We collected 455 spatial points and 329 foraging and anti-predator behavior observations on three flocks. The single best model for explaining the space use patterns of the flocks contained only vegetation density that surrounded Dusky-throated Antshrikes.

**Conclusion:**

The results are consistent with the hypothesis that a single species in mixed-species flocks has a disproportionately large influence on space use patterns. The surrounding vegetation density of the Dusky-throated Antshrike was the only driver of space use patterns of flocks supported by our data. The results may apply to flocks pantropically, many of which are led by species that behave similarly to the Dusky-throated Antshrike, e.g. Asian flocks led by drongos (*Dicrurus spp.*).

## Background

Determining where animals spend time is key for understanding their ecology and informing conservation efforts [7, 31, 39, 41, 54]. Animals may associate with conspecifics in groups, such as breeding pairs, herds, and colonies. The proximate drivers of associating with other individuals include mating opportunities, anti-predator defense, and increased foraging efficiency. For similar reasons (barring mating opportunities), animals may associate with individuals of different species. The drivers of space use of single individuals and monospecific groups are well studied; in contrast, few studies have examined drivers of space use of mixed-species groups [3, 20, 25, 60, 81], which are prevalent in avian communities [27, 30, 72].

Throughout the world, hundreds of species participate in mixed-species flocks [8, 29, 55]. A mixed-species flock is a group of individual birds that move spatially and independent of each other, although in the same direction [26]. Recent studies have focused on flock composition [4, 82], physical and behavioral similarities among flock participants [72–74], the stability of flocks over time [45], and the effect of urbanization on flock dynamics [27, 44]; few studies, however, have addressed factors that influence their space use (but see [60]).

Organisms prefer to spend time in areas rich in resources and low in predation risk [19, 23, 32, 33]. For example, Swainson’s Warblers (*Limnothlypis swainsonii*) space use corresponded to the patchy spatial distributions of certain plants and habitat types [3]. In single-species groups, the decisions of space use are typically made by one or a few individuals rather than collectively by all individuals [49, 58, 79]. Movements of olive baboons (*Papio anubis*) are initiated by a single individual when that individual moves in a highly directed manner [76]. In wintering groups of unrelated Willow Tits (*Poecile montanus*), adults are more likely to lead than hatch-year birds [35].

A mixed-species flock may function similarly to a single-species group in that a single species or individual may largely decide space use of the entire flock. In Amazonian mixed-species flocks, the Dusky-throated Antshrike (*Thamnomanes ardesiacus*; hereafter Dusky-throated Antshrike) and the Long-winged Antwren (*Myrmotherula longipennis*; hereafter Long-winged Antwren) are nuclear species, which are species nearly always found in a flock, and almost never away from a flock [30, 53]. Some nuclear species are virtually always found in flocks, but not all flocks contain those species, e.g. Bluish-slate Antshrike (*T. schistogynus*) and White-flanked Antwren (*M. axillaris*). Over 50 other transient species, e.g. White-eyed Antwren (*Epinecrophylla leucophthalma*) and White-flanked Antwren (*Myrmotherula axillaris*), may associate with these flocks or not [17, 38, 53, 79]. Because transient species are found in the flocks inconsistently, we did not expect transient species to have a regular influence on space use of flocks. Other flock participant roles include: leader species, which facilitate flock formation and cohesion; follower species, which follows other individuals in the flock; and sentinel species, which detect and alert the presence of predators. Some species simultaneously may play multiple roles, such as the Dusky-throated Antshrike, which is considered a nuclear, a leader, and a sentinel.

The distribution of animals can often be predicted by habitat characteristics [6, 24, 68], which suggests that animals rely on habitat characteristics when making fine-scale space use decisions. For example, animals likely use vegetation density as an indicator of resource availability and predation risk, although the preferred vegetation density is highly species-specific [14, 56, 67]. Dusky-throated Antshrikes scan leaves from an exposed perch for arthropods at a height of 2.6 m above the ground. Upon detecting prey, they sally-glean the surfaces of leaves, whereby stationary prey is removed from a leaf in flight. They return to their perch and dismantle their prey. This preference for exposed perches, i.e. sparsely vegetated areas, and sally-gleaning foraging style yield opportunities to simultaneously scan for distant ambush predators like forest-falcons [69, 80]. Dusky-throated Antshrikes likely spend time in areas with a low vegetation density because there are available prey and a low risk of predation. Transient flock species are attracted to Dusky-throated Antshrikes more strongly than to Long-winged Antwrens [83]. Therefore, the space use patterns of the flock may be driven, in large part, by the preferred habitat characteristics of the Dusky-throated Antshrikes.

We hypothesized that the foraging attack rate (a proxy for resource availability [59, 63];), vigilance rate (a proxy for predation risk [13, 47];), and vegetation density surrounding the Dusky-throated Antshrikes would explain the space use of the flock. We predicted that Dusky-throated Antshrikes would have higher attack rates, lower vigilance rates, and forage in sparser vegetation density in high use areas of the flock compared to low-use areas. Conversely, we predicted that the attack rates, vigilance rates, and vegetation density of the other nuclear, less attractant species, i.e. Long-winged Antwren, Bluish-slate Antshrike, and White-flanked Antwren, would not be different in high and low use areas of the flock. If the data support these predictions, it suggests that high use areas of the flock are optimal for the Dusky-throated Antshrike rather than the other species, and that Dusky-throated Antshrike preferences drive space use of the flock.

## Methods

### Study sites

Data were collected at Los Amigos Biological Station (12.568 S, 70.100 W) in May-Aug 2013–2014, during the dry season when breeding activity is relatively low. Los Amigos is situated among 1500 km^2^ of primary rainforest at 300 m above sea level in eastern Madre de Dios, Peru. Understory mixed-species flocks containing antshrikes and antwrens are abundant and present year-round, and are located by listening for the continuous vocalizations of flock members. Over 50 species participate in these mixed-species flocks [53]. Based on our observations from 2012 to 2015, the home ranges of the flocks are stable, and neighboring flocks have relatively little overlapping area (<5% of total home range size), consistent with previous work [45]. In May-Jul 2013 and May-Jun 2014, SMW color-banded 26 individuals of four species (Dusky-throated Antshrike, Bluish-slate Antshrike, Long-winged Antwren, and White-flanked Antwren) from three flocks in terra firme habitat for individual recognition. Two of the flocks were adjacent to each other and their home ranges overlapped by about 50 m^2^. Another flock was 650 m away from these two flocks and did not overlap.

### Spatial data collection

Data were collected on each flock 1–2 times per week. We entered each home range 5 minutes before dawn at a location where the flock was known to gather every morning. We followed a flock until 6 hours after dawn. We considered birds to be flocking when they were actively moving along branches and gleaning or sallying insects, and maintaining a distance of 10 m or less between species for five or more minutes [51, 75]. We observed birds at a distance of 1–10 m from the bird when birds were unobstructed by vegetation. Distance was judged based on visual calibration after practice with rangefinders and measuring tape. Every twenty minutes we took geographic coordinates of the flock using a Garmin GPSMap 78, which was accurate to 3–5 m [3]. The coordinates were taken at the position judged to be the centroid of the flock such that all or nearly all flock members were within 5 m of the point.

### Behavioral observations

We recorded behavioral observations of antshrikes and antwrens as long as possible (min 30 s) using a digital voice recorder while the birds were foraging. Flocks were followed each day, and behavioral data was collected opportunistically when birds remained in view for 30 s or longer. We recorded the vegetation density, the attack rate, the vigilance rate, and GPS coordinates of the position of the bird for every behavioral observation, following the focal-animal sampling method proposed by Altmann [1].

No more than one observation per hour per color-banded individual was taken in order to reduce non-independence of observations, following recommendations by Swihart & Slade [78], Lair [42], and Pechacek [57]. When collecting observations on non-banded individuals, we did not use an individual of the same species more than once per hour unless we were certain it was known to be a different individual based on plumage differences due to age or sex. The flocks moved 120–180 m per hour, although they sometimes moved over 300 m per hour. The home ranges were less than 300 m at the widest, and so flocks could have moved to any point in the home range within an hour. Therefore, the movements over 1 hour reflect choices by the flocks to forage in preferred areas rather than an inability to reach any location within a home range [42].

The attack rate was defined as the number of capture attempts of prey per unit time. The attack rate approximates the number of insects consumed and so the attack rate increases with prey availability [36, 59, 63]. Following recommendations of Remsen & Robinson [62], we defined an attack as the action of the bill striking or picking up an object. Vigilance rate is the proportion of time spent vigilant,which has been used as a proxy of predation risk since vigilance increases with predation risk [13, 43, 47]. Because antshrikes and antwrens forage low to the ground (<5 m), and forest-falcons ambush from the canopy, birds were considered vigilant when the bill was held horizontally or pointed upward [48, 65, 69].

Immediately following an observation, we visually estimated the vegetation density within a one-meter-radius sphere of the bird based on the percent of light that passed through the sphere, following recommendations of “foliage density” sampling by Remsen & Robinson [62]. A score of 0% indicated that all light passed through the sphere because there was no vegetation.

Although we observed 32 species in the flocks, only four species were common enough to obtain a sufficient number of observations to be included in the analyses. The four species used in the analyses were: the Long-winged Antwren, the Dusky-throated Antshrike, the White-flanked Antwren, and the Bluish-slate Antshrike.

### Data analyses

The home range of the flock was defined as the 95% fixed-kernel home range, following recommendations of Worton [84]. A high use area was calculated using the inner 55% area from the modeled kernel distribution, and the low use area was defined as the area lying outside the high use area, but still within the home range. Isopleths of 50–60% are typically used for defining high use area [3, 34, 66]. The 55% isopleth was chosen because it delineated hotspots of space use (pers. obs.). The home ranges, high use areas, and low use areas were calculated with the “kernelUD” and “getverticeshr” functions of the “adehabitatHR” package of the R Statistical Software, version 3.2.3 [9, 12]. The smoothing parameters were chosen using least-squares cross-validation, following recommendations of Seaman et al. [70].

To determine whether enough locations were sampled for home range kernel density estimation, we used the “rhr” package of the R Statistical Software, version 3.2.3 [9, 71]. Home range asymptotes were reached for each flock after 50 locations, which is a typical number of sampling locations to reach an asymptote [2, 70].

We used a generalized mixed model with a binomial distribution and logit link function to investigate whether attack rate, vigilance rate, and vegetation density surrounding the bird, of each of the four species distinguished areas of high (within the 55% isopleths of the home ranges) or low (between the 55 and 95% isopleths of the home ranges) space use by the flocks. Flock and individual were considered random effects. We built a set of candidate models to investigate the effects of attack rate, vigilance rate, and vegetation density of all four focal species on the likelihood of presence of the flock (Table 4 in Appendix). Models included all combinations of attack rate, vigilance rate, and vegetation density of the four focal species (full model), the Dusky-throated Antshrike, the three non-Dusky-throated Antshrike focal species, and a set of models whereby the data of each of the three behavioral variables were not coded by species. We calculated an Akaike Information Criterion (AIC), ΔAIC (AIC_*i*_–AIC_*min*_), and normalized model likelihoods (*w*) for each model.

The best selected model was at least 2 AIC units lower than the model with the next lowest AIC score [11]. The directions and strengths of the effects of the predictor variables on the response variable were estimated with 95% confidence intervals. A strong effect was defined as an interval that did not include zero, an intermediate effect included zero but was not centered on zero, and a non-effect was centered on zero [10, 18]. The “glmer” function of the “lme4” package was used for modeling [5] and the “AICtab” function of the “bbmle” package was used for the model selection [9]. All analyses were performed with the R Statistical Software, version 3.2.3 [9, 61]. The estimates are reported ± standard errors.

## Results

**Fig. 1:**
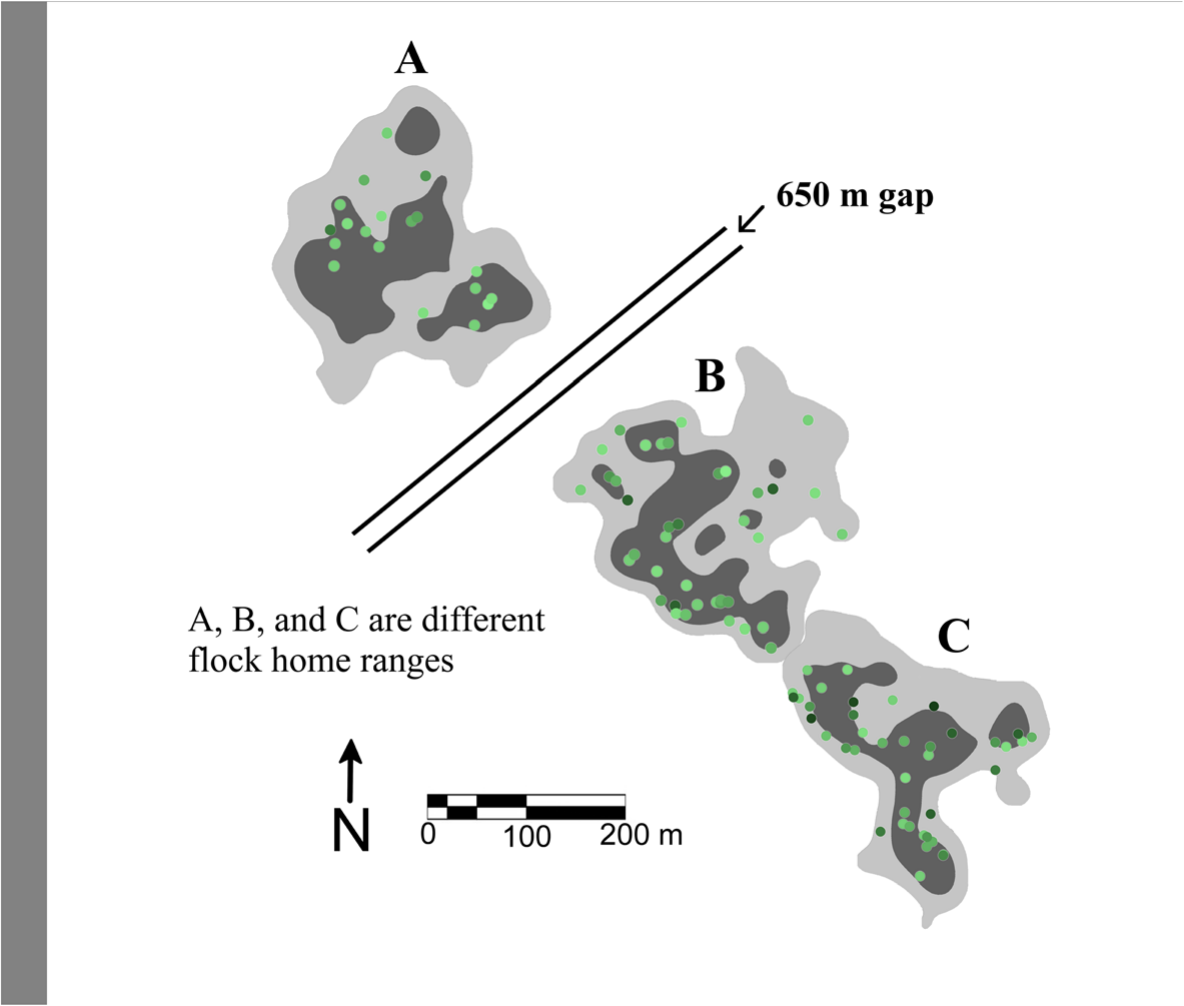
Home ranges (light gray plus dark gray areas), high use areas (dark gray areas), and low use areas (light gray areas) of three mixed species flocks (**a**, **b**, and **c**) at Los Amigos Biological Station, Peru in May–August 2014. The differently shaded green dots illustrate the vegetation densities recorded for Dusky-throated Antshrikes. A lighter green indicates a sparser vegetation density (minimum 0%) and a darker green indicates a denser vegetation density (maximum 65%). According to our prediction, there should be a higher proportion of darker green dots in the light gray area compared to the dark gray area. The double black lines indicate a spatial gap of 650 m

We collected, 134, 173, and 148 locations for each of the three flocks. Home range sizes were 6.74, 6.34, and 5.08 ha, and high use areas were 2.04, 2.46, and 1.79 ha, respectively (A, B, and C of Fig. 1). We collected 329 behavioral observations from 26 individuals of four species (Table 1). 288 of the observations (83%) came from banded individuals. A total of 32 species joined the flocks (Table 5 in Appendix).

**Table 1:** Behavioral observations from the four most common species found in three understory flocks at Los Amigos Biological Station, Madre de Dios, Peru in 2014

Species	Number of individuals	Total number of observations	% points in high use areas	% points in low use areas
Dusky-throated Antshrike	7	104	65	35
Bluish-slate Antshrike	4	38	34	66
Long-winged Antwren	9	139	63	37
White-flanked Antwren	6	48	50	50

Only one model was substantially supported (< 2 ΔAIC), and this model (*w* = 0.53) contained vegetation density of the Dusky-throated Antshrike as the only fixed-effect predictor (Table 2). The 95% confidence interval of the effect of vegetation density of the Dusky-throated Antshrike on the space use of the flock was (− 8.53, − 2.07). The ΔAIC of the next two most likely models both were 2.0 and included the vegetation density of the Dusky-throated Antshrike and either the attack rate (− 30.36, 34.58) or vigilance rate (− 14.26, 15.69) of the Dusky-throated Antshrike. The mean vegetation density of the Dusky-throated Antshrike was lower in the high use areas of the flock than low use areas (Table 3). None of the models with ΔAIC < 2 contained individual or flock effects.

**Table 2:** Results of model selection for the ten models with the smallest ΔAIC plus a null model

Model	ΔAIC	*K*	*w*
veg. DTAN	0	4	0.53
veg. DTAN + at. DTAN	2.0	5	0.20
veg. DTAN + vig. DTAN	2.0	5	0.20
veg. DTAN + at. DTAN + vig. DTAN	4.0	6	0.073
vig. DTAN	10.9	4	0.0023
at. DTAN	11.2	4	0.0020
at. DTAN + vig. DTAN	12.8	5	0.00
at. BSAN + at. WFAN + at. LWAN	180.0	6	0.00
veg. BSAN + veg. WFAN + veg. LWAN	180.3	6	0.00
vig. BSAN + vig. WFAN + vig. LWAN	180.8	6	0.00
	380.3	3	0.00

The models relate behavioral variables of individual flock members to the space use of the entire flock. “veg” is the vegetation density, “at” is the attack rate, and “vig” is the vigilance rate. DTAN stands for Dusky-throated Antshrike, BSAN stands for Bluish-slate Antshrike, WFAN stands for White-flanked Antwren, and LWAN stands for Long-winged Antwren. We define ΔAIC as the minimum AIC subtracted from the AIC of the corresponding model; *K* is the number of included parameters; and *w* is the normalized model likelihood [11]

**Table 3:** Means and standard deviations for the three variables of all four species included in the model

Variable	Species	High use mean ± SD	Low use mean ± SD
Vegetation density	Dusky-throated Antshrike	0.140 ± 0.102	0.249 ± 0.188
	Bluish-slate Antshrike	0.201 ± 0.200	0.132 ± 0.101
	Long-winged Antwren	0.482 ± 0.193	0.505 ± 0.190
	White-flanked Antwren	0.421 ± 0.2085	0.423 ± 0.201
Attack rate	Dusky-throated Antshrike	0.0131 ± 0.0149	0.0128 ± 0.00949
	Bluish-slate Antshrike	0.0230 ± 0.0271	0.0151 ± 0.0136
	Long-winged Antwren	0.0285 ± 0.0184	0.0282 ± 0.0226
	White-flanked Antwren	0.0196 ± 0.0180	0.0241 ± 0.0191
Vigilance rate	Dusky-throated Antshrike	0.937 ± 0.0294	0.935 ± 0.03040
	Bluish-slate Antshrike	0.946 ± 0.0449	0.963 ± 0.0258
	Long-winged Antwren	0.0485 ± 0.117	0.0356 ± 0.0756
	White-flanked Antwren	0.0394 ± 0.0835	0.0192 ± 0.0488

The Dusky-throated Antshrike had a lower mean vegetation density in the high use areas of the flocks relative to the low use areas

## Discussion

The results supported our prediction that the surrounding vegetation density of the Dusky-throated Antshrike, explained space use patterns of mixed-species flocks. None of the other models containing variables for the other species were supported. The areas in which the flocks spent the most time could be predicted by vegetation density of Dusky-throated Antshrike foraging locations.

Dusky-throated Antshrikes forage by perching still in areas with low vegetation density and scanning distant vegetation [69, 80]. They search for food hyperopically, which likely facilitates the detection of aerial predators, i.e. forest-falcons, which commonly barrage flocks. Dusky-throated Antshrikes give loud alarm calls upon the detection of the forest-falcons. Their alarm call is a likely mechanism for the strong attraction of antwrens and transiently flocking species to Dusky-throated Antshrikes. The attraction of Dusky-throated Antshrikes to Long-winged Antwrens is relatively weak, although the reciprocal attraction is strong [46, 83]. The nearly identical space use patterns of Dusky-throated Antshrikes and Long-winged Antwrens likely is due to this strong attraction (Table 1). Dusky-throated Antshrikes likely move to, and spend time in, areas with low vegetation density since, from the perspective of the Dusky-throated Antshrike, there are accessible prey and low predation risk. Other species then follow the Dusky-throated Antshrikes to gain anti-predation benefits [46]. Therefore, the space use patterns of Dusky-throated Antshrikes drive, at least in part, the space use patterns of the whole flock.

We predicted that Dusky-throated Antshrikes would prefer to forage in areas with high foraging efficiency and low predation risk. The models containing attack rate and vigilance rate of the Dusky-throated Antshrike yielded the next best levels of support. Attack rate and vigilance rate may both be correlated with vegetation density, if Dusky-throated Antshrikes select areas with the preferred vegetation density. However, using Pearson’s product-moment correlation test, there was no significant positive or negative association between vegetation density and either attack rate or vigilance rate.

Although predation risk and resource availability frequently are associated with space use patterns, other mechanisms, such as competition, may play a role in space use. Neotropical insectivorous birds, i.e. antbirds, defend territories against floater individuals or territory intruders [22, 50]. Because antbirds are highly territorial, it is possible that Dusky-throated Antshrikes spend time in areas with sparse vegetation in order to visually search for and chase away territory intruders [77]. If this hypothesis is correct, the simulated presence of intruding conspecifics (e.g. through playback) should cause Dusky-throated Antshrikes to forage in sparsely vegetated areas more frequently than control Dusky-throated Antshrikes.

Alternatively, we recognize that the behavioral variables we measured–attack rate and vigilance rate–are proxies for available food resources and predation risk, and therefore are imperfect measures of real-time food resource availability and predation risk [37, 63]. It is possible that the actual success rate of capturing prey items and the actual predation risk were higher and lower, respectively, in the low vegetation areas compared to the high-vegetation areas, but that our measures could not capture these differences.

Another species of antshrike, the Bluish-slate Antshrike, has been considered to perform a similar role to the Dusky-throated Antshrike in mixed-species flocks; they give raucous alarm calls in the presence of predators and are thought to be leaders of some flocks [38, 52, 53]. Bluish-slate Antshrikes occurred in two flocks intermittently. On several occasions (n ~ 20), the Bluish-slate Antshrike departed from the flock and continued foraging in other parts of the home range. Whenever the Bluish-slate Antshrike departed from the flock, the White-flanked Antwrens also disappeared and on the three occasions we pursued the Bluish-slate Antshrikes, we found the White-flanked Antwrens and Bluish-slate Antshrikes associated with each other. It is possible that the Bluish-slate Antshrike plays a role similar to the role of the Dusky-throated Antshrike and influences flock space use disproportionately for other flock species.

## Conclusion

The vegetation density surrounding the Dusky-throated Antshrikes was the best predictor of high and low use of the home range of the flock. Other species around the world may dictate space use patterns of mixed-species flocks similar to the Dusky-throated Antshrikes. Such species include Orange-billed Babblers (*Turdoides rufescens*) and Greater Racket-tailed Drongos (*Dicrurus paradiseus*) in Sri Lanka, Square-tailed Drongos (*Dicrurus ludwigii*) in Tanzania, Buff-rumped Thornbills (*Acanthiza reguloides*) in Australia, and Gray-cheeked Fulvettas (*Alcippe morrisonia*) in Taiwan [15, 16, 21, 40]. Drongos visually scan for insects in sparsely vegetated areas, similar to the Dusky-throated Antshrike, and so flocks led by drongos may spend a disproportionate amount of time in sparsely vegetated areas relative to the available vegetation densities [28, 64]. Some species may serve only as indicators of a flock’s presence and not confer foraging or anti-predation benefits to flocking species, e.g. the Orange-billed Babbler [28]. These indicator species may not drive space use patterns of the flock since transient species, after having found the flock, would experience no benefit from following the indicator species after they have joined the flock. Future studies should aim to predict habitat variables that are important to beneficial species, and investigate whether the space use patterns of the flock are associated with those habitat variables across the flock’s home range.

## Data Availability

The data are available online with the supplementary materials of this publication.

## Appendix

**Table 4:** The surrounding vegetation densities (veg), attack rates (at), and vigilance rates (vig) of Dusky-throated Antshrikes (DTAN), Bluish-slate Antshrikes (BSAN), White-flanked Antwrens (WFAN), and Long-winged Antwrens (LWAN) were included in a full model. Reduced models included different combinations of the four focal species and three behavioral data types based on different predictions of the four species and behavioral variables

Models included in analyses	
DTAN (at+vig + veg) + BSAN (at+vig + veg) + LWAN (at+vig + veg) + WFAN (at+vig + veg)	
DTAN (at+vig) + BSAN (at+vig) + LWAN (at+vig) + WFAN (at+vig)	
DTAN (at+veg) + BSAN (at+veg) + LWAN (at+veg) + WFAN (at+vig)	
DTAN (vig + veg) + BSAN (vig + veg) + LWAN (vig + veg) + WFAN (vig + veg)	
DTANat+BSANat+LWANat+WFANat	
DTANvig+BSANvig+LWANvig+WFANvig	
DTANveg+BSANveg+LWANveg+WFANveg	
At+vig + veg	
At+vig	
At+veg	
Vig + veg	
At	
Vig	
Veg	
DTAN (at+vig + veg)	
DTAN (at+vig)	
DTAN (at+veg)	
DTAN (vig + veg)	
DTANat	
DTANvig	
DTANveg	
BSAN (at+vig + veg) + LWAN (at+vig + veg) + WFAN (at+vig + veg)	
BSAN (at+vig) + LWAN (at+vig) + WFAN (at+vig)	
BSAN (at+veg) + LWAN (at+veg) + WFAN (at+vig)	
BSAN (vig + veg) + LWAN (vig + veg) + WFAN (vig + veg)	
BSANat+LWANat+WFANat	
BSANvig+LWANvig+WFANvig	
BSANveg+LWANveg+WFANveg	

**Table 5:** List of participating species found in mixed-species flocks at Los Amigos Biological Station May-Aug 2014. Nuclear species are always or nearly always found in all or nearly all flocks. Common transients are found in at least some flocks at least some of the time, and uncommon transients are found in few flocks some of the time

Nuclear	Common transient/semi-nuclear	Uncommon transient
Dusky-throated Antshrike (*Thamnomanes ardesiacus)*	Bluish-slate Antshrike (*Thamnomanes schistogynus*)	Fasciated Antshrike (*Cymbilaimus lineatus*)
Long-winged Antwren (*Myrmotherula longipennis*)	White-eyed Antwren (*Epinecrophylla leucophthalma*)	Plain-throated Antwren (*Isleria hauxwelli*)
	Madeira Antwren (*Epinecrophylla amazonica*)	Spot-winged Antshrike (*Pygiptila stellaris*)
	White-flanked Antwren (*Myrmotherula axillaris*)	Ornate Antwren (*Epinecrophylla ornata*)
	Gray Antwren (*Myrmotherula menetriesii*)	Sclater’s Antwren (*Myrmotherula sclateri*)
	Elegant Woodcreeper (*Xiphorhynchus elegans*)	Pygmy Antwren (*Myrmotherula brachyura*)
	Buff-throated Woodcreeper (*Xiphorhynchus guttatus*)	Cinnamon-rumped Foliage-gleaner (*Philydor pyrrhodes*)
	Rufous-rumped Foliage-gleaner (*Philydor erythrocercum*)	Rufous-tailed Foliage-gleaner (*Anabacerthia ruficaudata*)
	Olive-backed Foliage-gleaner (*Automolus infuscatus*)	Amazonian Barred-Woodcreeper (*Dendrocolaptes certhia*)
	Tawny-crowned Greenlet (*Tunchiornis ochraceiceps*)	Black-tailed Leaftosser (*Sclerurus caudatus*)
	Red-crowned Ant-Tanager (*Habia rubica*)	Olivaceous Woodcreeper (*Sittasomus griseicapillus*)
		Long-tailed Woodcreeper (*Deconychura longicauda*)
		Plain-brown Woodcreeper (*Dendrocincla fuliginosa*)
		Ocellated Woodcreeper (*Xiphorhynchus ocellatus*)
		Plain Xenops (*Xenops minutus*)
		Olive-striped Flyatcher (*Mionectes olivaceus*)
		Ochre-bellied Flycatcher (*Mionectes oleagineus*)
		Ruddy-tailed Flycatcher (*Terenotriccus erythrurus*)
		Musician Wren (*Cyphorhinus arada*)

## Abbreviations

BSAN: Bluish-slate Antshrike
DTAN: Dusky-throated Antshrike
LWAN: Long-winged Antwren
WFAN: White-flanked Antwren
